# Association between depression and osteoporosis in a population of cancer survivors: results from the NHANES 2005–2020

**DOI:** 10.3389/fmed.2025.1515435

**Published:** 2025-04-22

**Authors:** Shuchen Hou, Fengquan Xu, Guanhua Zong, Yu Zheng, Lei Shi, Liangfan Zhai

**Affiliations:** ^1^Graduate School, Beijing University of Chinese Medicine, Beijing, China; ^2^Guang’anmen Hospital, China Academy of Traditional Chinese Medicine, Beijing, China

**Keywords:** cancer survivor, depression, bone density, osteoporosis, cross-sectional study

## Abstract

**Introduction:**

Depressive symptoms and the severity of osteoporosis in cancer survivors significantly affect the patient’s quality of life. The correlation between osteoporosis and depressive symptoms in this population has not been examined in prior studies. This cross-sectional study utilized National Health and Nutrition Examination Survey data from 2005 to 2020 to explore the correlation between osteoporosis and depressive symptoms in cancer patients.

**Method:**

We utilized confounder-adjusted multivariate logistic regression models to examine the relationship between osteoporosis and depressive symptoms. Subgroup and interaction analyses were performed according to age and gender to detect potential differences among various demographic groups. Furthermore, smoothing curve fitting and subgroup smoothing curve fitting were employed to evaluate the nonlinear association between bone mineral density and depressive symptom scores.

**Results:**

Two thousand one hundred and fifty-five adult cancer patients satisfied the inclusion and exclusion criteria and were recruited in the research. Statistical analysis demonstrated a significant negative correlation between depressive symptoms and the risk of developing osteoporosis in cancer patients after controlling for multiple variables [OR 0.57, 95% CI (0.46–0.70), *p* < 0.01]. Subgroup analyses revealed more pronounced associations in women [OR 0.18, 95% CI (0.12–0.27), *p* < 0.01] and older adults [OR 0.09, 95% CI (0.06–0.13), p < 0.01]. Furthermore, smooth curve fitting results displayed a W-shaped curve between bone mineral density and depressive symptom scores. This W-shaped curve association was especially prominent among older patients in subgroup analyses.

**Discussion:**

This study demonstrates a negative correlation between depressive symptoms and the incidence of osteoporosis in cancer survivors, notably evident among the elderly and female populations. Our research addresses the relationship between depressed symptoms and osteoporosis in cancer survivors, revealing a negative association that may alleviate psychological distress and enhance the quality of life in this population.

**Conclusion:**

A negative correlation between depressive symptoms and osteoporosis in cancer patients, particularly pronounced in the elderly and female survivors. Additionally, a W-shaped relationship was observed between bone mineral density and depression scores, with greater significance in the older group.

## 1 Introduction

Osteoporosis, abbreviated as OP, is a common disease of the bones primarily characterized by a significant decrease in the mass of the bones throughout the body and irreversible destruction of the bone microstructure. This elevates bone fragility and the likelihood of fractures, even from modest falls or impacts. Individuals with OP are at a high risk of fractures due to this pathological change (1, 2). As the world population ages, osteoporotic fractures are becoming increasingly prevalent and adversely affecting individuals’ quality of life (3). The global incidence of OP is estimated to be around 18.3%. Regarding gender inequalities, the prevalence of OP was 11.7% in males and 23.1% in women. The incidence in undeveloped countries is 22.1%, but in affluent nations, it is 14.5% (4). Considering that OP predominantly impacts older women and healthcare expenses are rising in several low-income nations, research on OP in this demographic possesses heightened therapeutic significance (5). In recent years, due to the ongoing advancement of molecular biology, theories of cellular senescence (6–8), intestinal flora and bone immunity disorder (9, 10), osteoblast energy metabolism, iron homeostasis, and iron death (11) have played a crucial role in the study of its pathogenesis.

Depression is a mental disorder characterized by persistent sadness, loss of interest or pleasure in activities, and impairments in daily functioning that significantly impact a person’s quality of life and career (12). According to a World Health Organization (WHO) survey, around 5% of adults worldwide suffer from depression (13). There is no definite conclusion on the many factors that contribute to the development of depression, such as psychological factors, social pressure, dietary habits, and genetic factors (14). It has been reported that only 3.6% of patients with depression receive specialized antidepressant treatment (15); hence, studying depression can significantly improve people’s quality of life. There is a correlation between depression and the development of OP. Beyond limiting physical activity due to complications such as fractures and chronic pain, OP can also manifest as a psychosomatic illness, affecting patients emotionally. Studies have shown that patients with depression have a heightened chance of acquiring OP, implying that depression may contribute to its etiology (16, 17). A comprehensive and nationally representative study done in the United States identified a link between depression and OP in the older population (18). Furthermore, depression may result in metabolic abnormalities due to neurohormonal imbalances. A stronger correlation has been observed between OP and depressive symptoms in postmenopausal women. The prevalence of concomitant OP is twice as high in those with depression compared to those without depressive symptoms. The correlation between depressed symptoms and OP in postmenopausal women is notably robust (19). The rapid loss of bone mass caused by a decline in estrogen levels during menopause contributes to OP development in women (20, 21). Investigating the connection between depression symptoms and OP is beneficial for both the diagnosis and treatment of OP.

Cancer is a significant public health issue in the twenty-first century, contributing to approximately 1/6 of all fatalities and 3/10 of premature deaths (30–69 years old) worldwide (22, 23). It also has a significant social and financial cost. The idea of cancer-related depression has drawn the attention of some experts and scholars. This is related to the pressures imposed on the patient throughout their illness because of their diagnosis of cancer and the treatment they are undergoing (24). Cancer survivors with depressive symptoms who then experience OP fractures or bone pain are likely to choose suicide (25).

Additionally, medications used to treat cancer, such as letrozole and other aromatase inhibitors, increase the risk of OP in cancer patients (26). Consequently, it is imperative to examine the correlation between depressive symptoms and OP in cancer patients since many of these individuals now contend with both conditions. This study analyzed data from a large, nationally representative U.S. population to investigate the relationship between depressed symptoms and OP in cancer patients, aiming to enhance their quality of survival.

## 2 Materials and methods

### 2.1 Study population in NHANES

This study employed data from the National Health and Nutrition Examination Survey (NHANES) administered by the National Center for Health Statistics (NCHS). NHANES data collection is conducted at all the United States locations. NHANES gathers extensive data about the health and nutritional condition of individuals in the United States using questionnaires, laboratory analyses, and physical assessments. All participants granted informed consent, and the NCHS Research Ethics Review Board sanctioned the protocols for the NHANES study. Our study employed cross-sectional data from participant questionnaires, laboratory results, physical exams, and dietary information. For additional details on the NHANES study design and data collection, please refer to the official website: http://www.cdc.gov/nchs/NHANES/. The study was designed following the guidelines of the STROBE Epidemiology Report.

**Figure 1:**
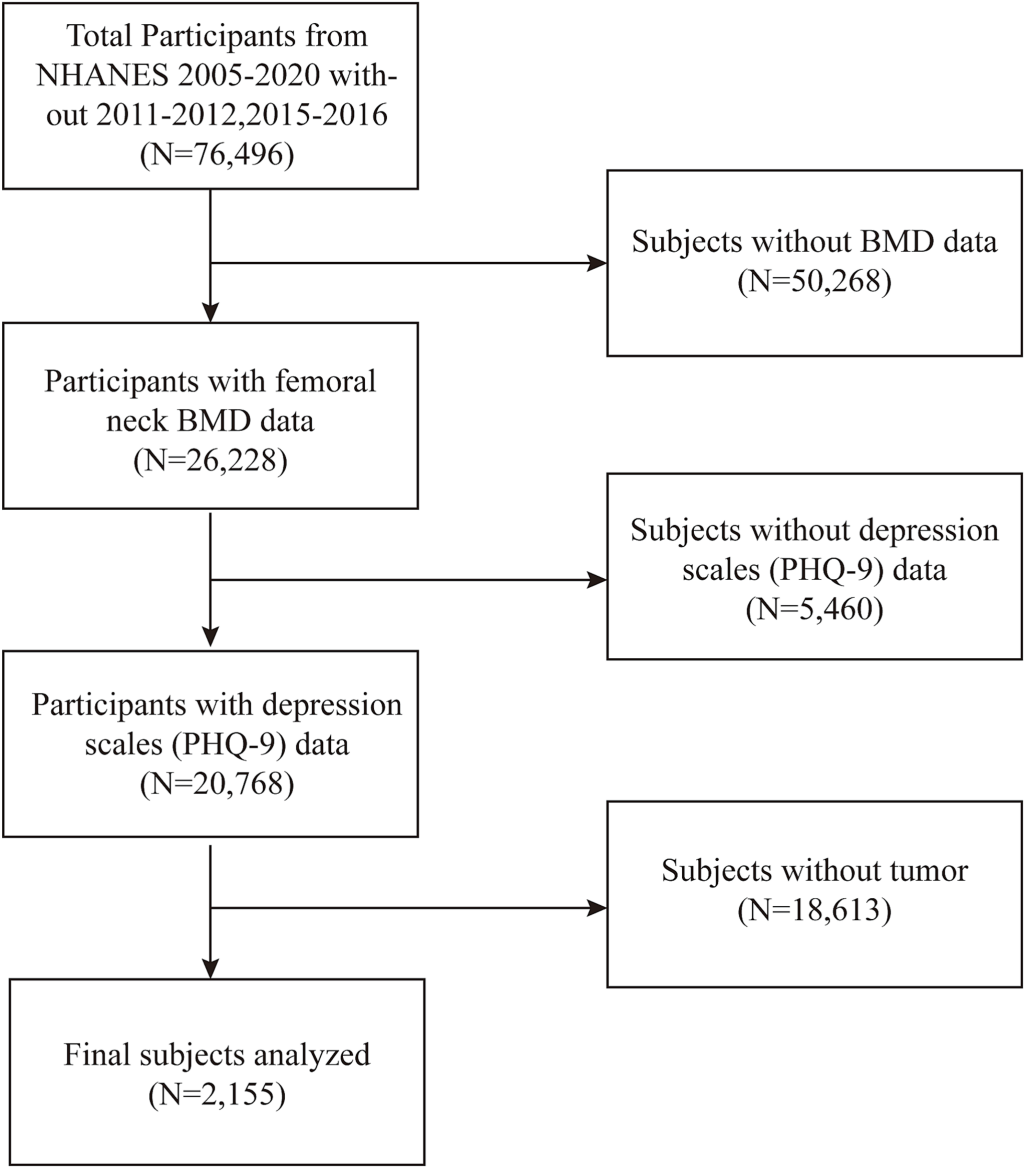
A flowchart of the data integration and analysis process.

We analyzed participant data collected from 2005 to 2020, with the 2017–2020 data representing pre-pandemic data gathered from 2017 to March 2020. Data from 2011–2012 and 2015–2016 were excluded due to incomplete femoral neck bone mineral density (BMD) measurements. Bone density data were indispensable as an outcome variable in our study. OP diagnosis was determined by dual-energy X-ray absorptiometry (DXA)-measured femoral neck bone mineral density (BMD). In this study, femoral neck BMD was the primary outcome variable, with BMD values below 0.556 g/cm^2^ classified as OP (27). Depression was assessed as the exposure variable, with a total PHQ-9 score ≥ 5 indicating depressive symptoms. The study population for this study was cancer survivors. Participants who answered “yes” to the MCQ220 questionnaire section on whether they had ever been told by a doctor that they had cancer were included in the study. In MCQ230a, participants answered specific types of cancer; therefore, the NHANES study covered the full range of cancer types. We removed all incorrect values and missing values. In the initial sample of 76,496 participants, 50,268 were excluded due to missing BMD data, 5,460 due to missing depression scale scores, and 18,613 due to the absence of a cancer diagnosis. Ultimately, the final analysis comprised 2,155 individuals (Figure 1).

### 2.2 Measures

The World Health Organization defines OP using the T-score of BMD calculated as (BMD-reference BMD)/reference standard deviation (SD). This research categorized individuals with a femoral neck BMD below 0.556 g/cm^2^ as having OP. Depressive symptoms were evaluated with the Patient Health Questionnaire-9 (PHQ-9), a nine-item measure for assessing depression. A score of ≥5 on the PHQ-9 was utilized to signify the existence of depressive symptoms, with elevated scores corresponding with a heightened probability of depression. Factors including low level of education, poor marital status, and low income may all contribute to depressed mood or reduced nutritional supplementation in patients, thereby affecting the relationship between depressive symptoms and OP. Based on the experience of previous studies, the occurrence of depression and OP may be associated with body mass index (BMI) (28, 29), smoking status (30, 31), age (32, 33), gender (34, 35), race (34, 36), education (36, 37), marital status (38), household income to poverty ratio (PIR) (39), and diabetes status (40, 41). The study encompassed the following covariates: BMI, smoking status, age, gender, race, educational attainment, marital status, PIR, and diabetes status. We used multivariate logistic regression to adjust for covariates to reduce the effect of confounders on the results. Comprehensive measurement methods for these variables are available at http://www.cdc.gov/nchs/nhanes/.

### 2.3 Statistical analysis

In this cross-sectional research, we employed *t*-tests and chi-square tests to examine the baseline characteristics of patients with and without OP. The correlation between OP risk and depressive symptoms was assessed by three multivariate logistic regression models, with outcomes reported as odds ratios (OR) and 95% confidence intervals (CI). Model 1 did not incorporate any modifications for variables. Model 2 incorporated modifications for age, gender, race, educational attainment, marital status, family income-to-poverty ratio, and BMI. Model 3 was also adjusted for diabetes, smoking status, and moderate-intensity physical activity alongside the variables from Model 2. We conducted a subgroup stratified analysis of the relationship between depressive symptoms and OP in a population of cancer survivors based on age, equally divided into 4 groups (Q1–Q4) and gender (male and female). A smoothed curve fitting technique was employed to analyze the linear correlation between PHQ-9 scores and BMD. Statistical analyses were conducted utilizing R^1^ and EmpowerStats.^2^

## 3 Results

### 3.1 Baseline characteristics

This investigation involved 2,155 subjects in total, 132 of whom had an OP diagnosis and 2,023 of whom did not (132/2,023). The age distribution of the patients with and without OP was 74.89 ± 7.75 years and 65.85 ± 12.91 years, respectively. There were no significant differences in the components of the variables of physical activity, education, smoking status and prevalence of diabetes (*p* > 0.05). In contrast, age, gender, income, race, and marital status were significantly different between those who had OP and those without OP (*p* < 0.05) (Table 1).

**Table 1:** Characteristics of the study population based on osteoporosis in cancer survivors.

Characteristic	Osteoporosis		*p*-value
	Have (BMD < 0.556)	Not have (BMD ≥ 0.556)	
Number	132	2,023	
Age in years	74.89 ± 7.75	65.85 ± 12.91	<0.001
Ratio of family income to poverty	2.47 ± 1.36	2.93 ± 1.52	<0.001
BMI	24.45 ± 5.04	28.68 ± 5.60	<0.001
Gender			<0.001
Male	32 (24.24%)	1,036 (51.21%)	
Female	100 (75.76%)	987 (48.79%)	
Race			0.002
Mexican American	3 (2.27%)	137 (6.77%)	
Other Hispanic	7 (5.30%)	101 (4.99%)	
Non-Hispanic White	112 (84.85%)	1,403 (69.35%)	
Non-Hispanic Black	6 (4.55%)	288 (14.24%)	
Other race	4 (3.03%)	94 (4.65%)	
Education			0.469
Less than 9th grade	15 (11.36%)	163 (8.06%)	
9–11th grade	16 (12.12%)	206 (10.18%)	
High school graduate/GED or equivalent	33 (25.00%)	474 (23.43%)	
Some college or AA degree	38 (28.79%)	614 (30.35%)	
College graduate or above	30 (22.73%)	566 (27.98%)	
Marital status			<0.001
Married/living with partner	60 (45.45%)	1,281 (63.32%)	
Widowed/divorced/separated	67 (50.76%)	621 (30.70%)	
Never married	5 (3.79%)	119 (5.88%)	
Other status	0 (0.00%)	2 (0.10%)	
Smoke			0.255
Yes	60 (45.45%)	1,098 (54.28%)	
No	72 (54.55%)	923 (45.63%)	
Exercise			0.011
Yes	37 (28.03%)	817 (40.39%)	
No	92 (69.70%)	1,191 (58.87%)	
Do not know	3 (2.27%)	14 (0.69%)	
Diabetes			0.511
Have	21 (15.91%)	377 (18.64%)	
Do not have	108 (81.82%)	1,574 (77.81%)	
Do not know	3 (2.27%)	72 (3.56%)	

Baseline characteristics of the 2,155 cancer survivors. Mean ± SD of continuous variables: *p*-value calculated by weighted linear regression model (%); Categorical variables: *p*-value calculated by weighted chi-square test BMD bone mineral density.

### 3.2 Association between depression and OP in the cancer survivor population

We investigated the relationship between OP and depression in cancer patients utilizing three multivariate logistic regression models (Table 2). The findings showed that the risk of OP was reduced by 38% [OR 0.62, 95% CI (0.43, 0.90), *p* = 0.01] in depressed patients in model 1; in model 2, the risk was reduced to 48% [OR 0.60, 95% CI (0.47, 0.76), *p* < 0.01]; and in model 3, the risk was 57% [OR 0.57, 95% CI (0.46, 0.70), *p <* 0.01]. These findings reveal a negative correlation between depressive symptoms and OP in cancer patients, with the odds of getting OP among cancer patients being 57% lower in those with depression than in those without. Nevertheless, no causal relationship between the two diseases can be inferred.

**Table 2:** Association between depression and osteoporosis in the cancer survivor population.

Variable	Model 1 OR(95%CI) *P* value	Model 2 OR(95%CI) *P* value	Model 3 OR(95%CI) *P* value
(PHQ-9)			
<5	Reference	Reference	Reference
≥5	0.62 (0.43, 0.90) 0.0113	0.57 (0.37, 0.88) 0.0103	0.57 (0.37, 0.88) 0.0111

Model 1 adjusted: none. Model 2 adjusted for gender, age, race, marriage, educational attainment, household income to poverty ratio, and body mass index. Model 3 adjusted for sex, age, race, marriage, education, household income to poverty ratio, body mass index, smoking, moderate-intensity physical activity, and diabetes.

### 3.3 Subgroup analyses

**Figure 2:**
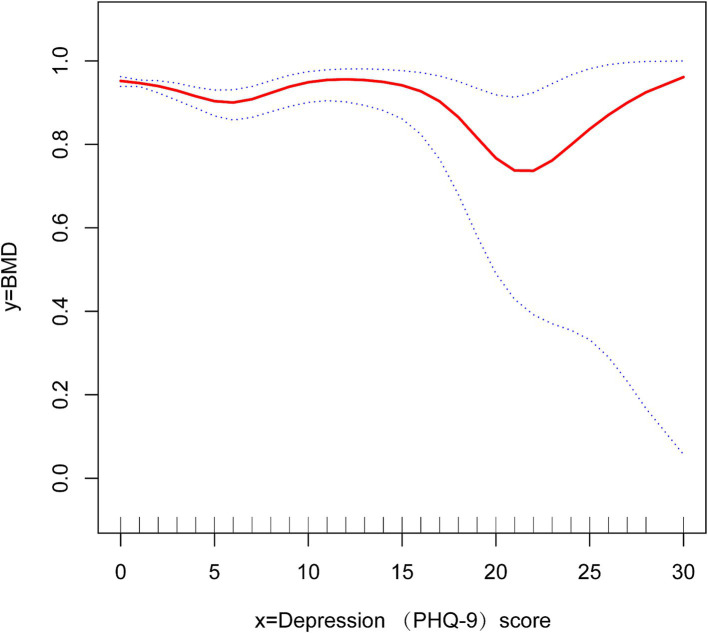
Smooth curve fitting reveals a w-shaped relationship between depression scores and bone density in cancer survivors.

**Figure 3:**
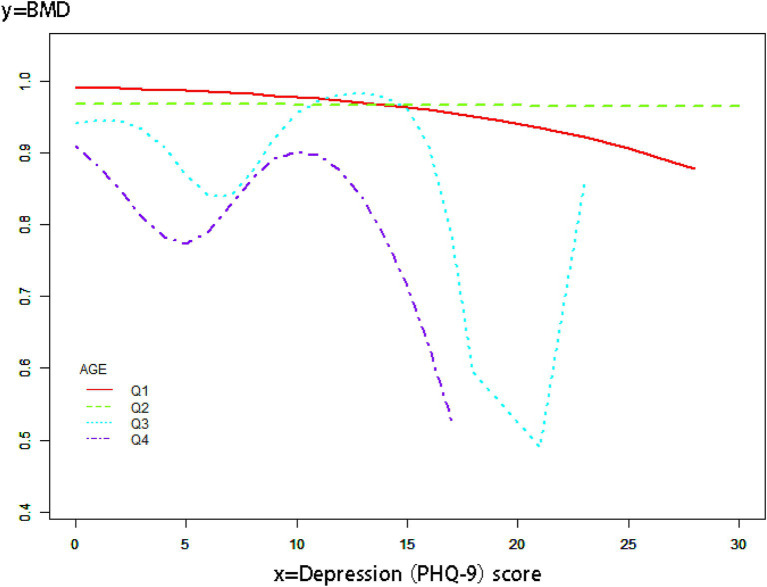
Smooth curve fitting of subgroup analyses describes the relationship between depression scores and BMD in cancer survivors of different age groups.

We conducted logistic regression for additional covariates and grouped the continuous variables into four quartiles (Q1-Q4) to analyze the association between depressive symptoms and OP in cancer patients (Table 3). Our findings indicated that the negative association was more significant in women [OR 0.18, 95% CI (0.12, 0.27) *p* < 0.01] and older adults [OR 0.09, 95% CI (0.06, 0.13) *p* < 0.01]. In this investigation of cancer patients, smoothed curve fitting (Figure 2) showed a W-shaped relationship between depression score (PHQ-9) and BMD. Based on subgroup analyses, we stratified the cancer survivor population by age. We performed smoothed curve fitting for each subgroup (Figure 3) and found that the W-shaped relationship was more significant in the older survivor groups (Q3 and Q4).

**Table 3:** Subgroup analysis of the relationship between depression and osteoporosis in cancer survivors.

Variable	(PHQ-9)	OR(95%CI)	*P* value	*P* (interaction)
Sex				0.694
Men	<5	Reference = 1		
Men	≥5	0.91 (0.42, 1.97)	>0.05	
Women	<5	Reference = 1		
Women	≥5	0.18 (0.12, 0.27)	<0.01	
Age				0.3221
Q1	<5	Reference		
Q1	≥5	0.01 (0.00, 0.80)	0.03	
Q2	<5	Reference		
Q2	≥5	0.02 (0.00, 0.63)	0.01	
Q3	<5	Reference		
Q3	≥5	0.08 (0.03, 0.21)	<0.01	
Q4	<5	Reference		
Q4	≥5	0.09 (0.06, 0.13)	<0.01	

## 4 Discussion

Depressive symptoms in cancer survivors may lead to suicide when coupled with bone pain or fractures due to OP. The association between OP and depressed symptoms in cancer survivors has not been investigated before. Many contemporary epidemiological studies examining the relationship between depressed symptoms and OP have concentrated on the general and elderly populations. Prior research has indicated a potential bidirectional causal link between depressed symptoms and OP. A cross-sectional NHANES study identified a favorable correlation between depressed symptoms and OP (18). Numerous expert consensus studies, systematic reviews, and meta-analyses have established a strong positive link between depressed symptoms and OP throughout the whole population, identifying depression as a definitive risk factor for OP (42, 43). A research examining adverse reactions to alendronate within the Formulated Adverse Event Reporting System (FAERS) revealed that the prevalent OP medication alendronate was linked to heightened incidences of depression and anxiety (44). In the elderly population, depressive symptoms impair physical activity, exacerbating OP in older individuals with the condition (45). Female patients aged over 50 may constitute a distinct demographic. A clinical investigation has demonstrated a negative correlation between depressed symptoms and OP in women over the age of 50 (46).

Cancer survivor populations exhibit a higher prevalence of depressive symptoms compared to the general population, attributable to low survival rates, and are at an increased risk for suicide owing to physical distress (47). A significant consensus exists about the association between cancer and OP. Patients with breast and prostate malignancies are more predisposed to developing OP as a result of hormonal imbalances (48). OP-related bone pain in cancer survivors typically results in significant discomfort (49). OP may elevate the chance of pathological fractures, particularly in the hip, perhaps resulting in extended bed rest and the gradual deterioration of the patient. Extended bed rest progressively undermines the patient’s mental health and induces depressed symptoms (50). The co-morbidity of depression and OP in cancer survivors can be profoundly detrimental, perhaps resulting in severe repercussions that may drive the individual to contemplate suicide (51). Consequently, it is essential to examine the correlation between depressed symptoms and OP in cancer survivors.

This study discovered a substantial negative connection between depressed symptoms and OP among cancer survivors. The negative link was more evident in female patients, aligning with other research that indicated a negative correlation between depressed symptoms and OP in women over 50 years old. Cancer survivors exhibiting depressed symptoms had a markedly reduced incidence of OP compared to those without depressive symptoms [OR 0.57, 95% CI (0.46, 0.70), *p* < 0.01]. The precise pathways between depression and OP among cancer survivors remain unidentified. In cancer patients, particularly those with breast and prostate malignancies, hormonal imbalances are linked to the onset of OP (52). As cancer severity increases, the probability of depression rises, leading to more systematic treatment by an oncologist in a hospital setting, therefore establishing a negative link between depressive symptoms and OP in cancer survivors. Cancer survivors experiencing depressive symptoms frequently have less tolerance for external stimuli and are prone to exaggerating somatic feelings, resulting in heightened alertness about physical changes, which may lead patients to pursue BMD screening or anti-OP therapy (53). In late-stage cancer survivors, malignant circumstances influence BMD, leading to the emergence of depression symptoms. Consequently, physicians often prescribe anti-osteoporotic drugs, establishing a negative association between depressive symptoms and OP. Cancer survivors in the advanced stages of malignancy will experience a reduction in their BMD; patients often exhibit depressive symptoms, prompting physicians to recommend anti-OP pharmacotherapy, thereby establishing a negative correlation between depressive symptoms and OP (54). The negative link may be particularly evident in women owing to the substantial hormonal fluctuations during menopause.

A W-shaped correlation exists between BMD and depression ratings in populations of cancer survivors. The W-shaped association may result from a mix of hormonal and neurotransmitter influences. Chronic stress in mild to severe depression elevates hormone levels, which suppresses bone production and results in decreased BMD (55). In those with moderate to severe depression, tailored therapy, together with enhanced outdoor exercise and dietary intake, leads to a temporary improvement in BMD (56). Patients with severe depression may endure extended durations of bed rest and malnutrition resulting from eating problems, which can precipitate a further loss in BMD (57). The results we obtained indicate that the type W relationship is more prominent in the older demographic, maybe due to their greater availability to seek hospital care for antidepressants or anti-osteoporotic medications compared to younger individuals.

The benefits and contributions of this work are as follows: This study represents the first investigation into the correlation between depressed symptoms and OP among cancer survivors, enhancing OP studies within this demographic. Compared with other studies, this study has a large sample size and high reliability of results. Furthermore, to enhance the trustworthiness of the findings, this study was comprehensively adjusted for confounding variables that might influence the results. Subgroup analyses were performed to clarify the relationship between depressed symptoms and OP in cancer patients across various ages and genders. This study identified a W-shaped correlation between BMD and depression scores among cancer survivors through smoothed curve fitting. The relationship was particularly pronounced in the elderly demographic.

This study’s limitations encompass the challenge of establishing a direct causal association between depressed symptoms and OP in cancer patients due to its cross-sectional methodology. We intend to pursue case–control studies in the future to investigate the causal link between these two factors. The impact of various malignancies on the association between OP and depressed symptoms necessitates more comprehensive data about distinct cancer kinds; the relatively uniform NHANES cancer-related data fails to provide specific information on cancer severity and stage. Cancer treatments may influence the correlation between depressed symptoms and OP in cancer survivors, and the NHANES database lacks medication data for cancer survivors, so we cannot dismiss the potential impact of anticancer drugs on the findings. Our team aims to gather more comprehensive patient data in the future to further explore the correlation between OP and depressed symptoms in cancer survivors with varying severity and drug regimes. A comprehensive cohort research will be undertaken to examine the correlation between variations in depression scale scores and OP in cancer survivors, therefore establishing a causal link between depressed symptoms and OP in this population. Despite the study’s consideration of many features and adjustments for confounders, the influence of all confounding variables could not be entirely eradicated.

Depressive symptoms exhibited a negative correlation with OP in cancer survivors. Communicating this finding to patients may alleviate the stress associated with concurrent illnesses and indirectly offer psychological solace to cancer survivors. The negative correlation between depressive symptoms and OP in cancer survivors was more pronounced in female patients, whereas it was not significant in male patients. Therefore, greater attention should be directed toward male cancer survivors in efforts to prevent the co-morbidity of depressive symptoms and OP.

## 5 Conclusion

This study found that depressive symptoms were negatively correlated with OP in cancer patients, which may be related to the population of cancer patients, and this effect was more significant in the elderly population and the female population. There was a W-shaped relationship between BMD and depressive scores, and the W-shaped relationship was more significant in the elderly population.

## Data Availability

The original contributions presented in the study are included in the article/supplementary material, further inquiries can be directed to the corresponding author.

## Footnotes

^1^ http://www.r-project.org

^2^ http://www.empowerstats.com
